# MTG-Link: leveraging barcode information from linked-reads to assemble specific *loci*

**DOI:** 10.1186/s12859-023-05395-w

**Published:** 2023-07-14

**Authors:** Anne Guichard, Fabrice Legeai, Denis Tagu, Claire Lemaitre

**Affiliations:** 1 IGEPP, INRAE, Institut Agro, Univ Rennes, 35653 Le Rheu, France; 2 Univ Rennes, Inria, CNRS, IRISA, 35000 Rennes, France

**Keywords:** Local assembly, Gap-filling, Linked-reads, Barcode

## Abstract

**Background:**

Local assembly with short and long reads has proven to be very useful in many applications: reconstruction of the sequence of a *locus* of interest, gap-filling in draft assemblies, as well as alternative allele reconstruction of large Structural Variants. Whereas linked-read technologies have a great potential to assemble specific *loci* as they provide long-range information while maintaining the power and accuracy of short-read sequencing, there is a lack of local assembly tools for linked-read data.

**Results:**

We present MTG-Link, a novel local assembly tool dedicated to linked-reads. The originality of the method lies in its read subsampling step which takes advantage of the barcode information contained in linked-reads mapped in flanking regions. We validated our approach on several datasets from different linked-read technologies. We show that MTG-Link is able to assemble successfully large sequences, up to dozens of Kb. We also demonstrate that the read subsampling step of MTG-Link considerably improves the local assembly of specific *loci* compared to other existing short-read local assembly tools. Furthermore, MTG-Link was able to fully characterize large insertion variants and deletion breakpoints in a human genome and to reconstruct dark regions in clinically-relevant human genes. It also improved the contiguity of a 1.3 Mb *locus* of biological interest in several individual genomes of the mimetic butterfly *Heliconius numata*.

**Conclusions:**

MTG-Link is an efficient local assembly tool designed for different linked-read sequencing technologies. MTG-Link source code is available at https://github.com/anne-gcd/MTG-Link and as a Bioconda package.

**Supplementary Information:**

The online version contains supplementary material available at 10.1186/s12859-023-05395-w.

## Background

Local assembly consists in reconstructing a sequence of interest from a sample of sequencing reads without having to assemble the entire genome, which is time and labor intensive. This is particularly useful when studying a *locus* of interest, e.g. including regions linked to a phenotype, or including patterns of positive selection, clusters of rapidly evolving genes or genes involved in a relevant biochemical pathway [1–3]. Reconstructing the full *locus* sequence or haplotypes in a given individual or sample is particularly relevant in presence of structural polymorphism at the given *locus*. The Supergene P *locus* of the mimetic butterfly *Heliconius numata* illustrates this perfectly, since this 1.3 Mb *locus*—controlling the wing color pattern—hosts 3 polymorphic inversions [4, 5]. The Immunoglobulin V-D-J genes in vertebrate species are also an example of *loci* where their full sequence reconstruction is required for studying the remarkable diversity of the immunity cell receptors that is generated by a complex recombination mechanism [6]. Another application of local assembly is the characterization of sequences not present in the reference genome, either because they are unresolved sequences or gaps in the reference assembly (problem referred to as gap-filling in draft assemblies) or because they correspond to structural polymorphisms, such as novel insertions in re-sequenced individuals or reference-specific deletions. In such contexts, limiting the assembly to specific genomic *loci* has many benefits. First, it drastically reduces the computational resources and running time compared to a full *de novo* genome assembly. Second, it can also result in more accurate or more contiguous assemblies as it may be less impacted by genome-wide repeats.

Hence, it is important to develop methods that perform local assembly of specific *loci*. Here, we define local assembly as the *de novo* assembly of a specific region of the genome using the sequencing reads coming from whole genome shotgun sequencing. Local assembly differs from targeted or reference-guided assembly. In targeted assembly, a sequence already known, typically from a closely related species, is used to recruit reads of interest by mapping or to guide the assembly. We can for instance mention aTRAM [7, 8], TASR [9], SRAssembler [10] for short-read data, SLAG [11] for long-read data and AquilaSV [12] for linked-read data, as targeted assembly tools. On the contrary, in local assembly, the sequence to be assembled is not already known, nor is a closely related sequence, and an approximation of its length is also unknown. The only known information is its location on a genome and therefore its left and right flanking sequences. Most local assembly tools were designed for gap-filling a draft assembly; among others one can cite GapCloser from the SOAPdenovo suite [13], Sealer from the ABYSS suite [14], GAPPadder [15] for short-read data and LR_gapcloser [16], TGS-Gapcloser [17] and DENTIST [18] for long-read data.

Local assembly algorithms and performances depend on the sequencing technology used. Indeed, long-read data are better suited than short-reads to any *de novo* assembly problem. But long-read sequencing technologies, such as Pacific Biosciences and Oxford Nanopore, suffer from higher costs and may not be affordable for population re-sequencing studies. Short Illumina reads are still massively used for re-sequencing studies, but their short read size makes them ill suited for *de novo* assembly tasks. Linked-read technologies provide the long-range information, that is crucially missing in short reads. With these technologies, all short reads that have been sequenced from the same long DNA molecule (around 30–70 Kb) are tagged with a specific molecular barcode. Several linked-read technologies have been developed and commercialized, including the one from the 10$\times$ Chromium Genomics company, which initially popularized this technology [19], but also Single Tube Long Fragment Read (stLFR) [20], TELL-Seq [21] and Haplotagging [22]. Even if 10$\times$ Chromium Genomics company recently stopped producing such data, large volumes of data were produced using this technology and the three other more recent technologies are now routinely used. Low-cost, low-input and high-accuracy linked-read technologies have many applications: *de novo* genome assembly [23], haplotype identification [19], genome scaffolding [24–26] and structural variant calling [12, 27–30].

Concerning the assembly of specific *loci*, there is to our knowledge only one tool for linked-read data, AquilaSV [12], but it is primarily a Structural Variant caller that performs targeted or reference-guided assembly. In AquilaSV, only the reads mapped to a reference sequence of the target *locus* are used to obtain a phased diploid assembly of the *locus*, therefore it is unable to assemble large sequences absent from the reference genome. In the case of local assembly, where a reference sequence for the *locus* to be assembled is not required, there is thus currently no tool that uses the long-range information of the linked-read data, although several such tools have been developed for short-read data. Short-read local assembly tools can be classified in two categories, depending on whether they use the whole set of reads for the assembly graph traversal or whether they rely on a first step of read recruitment. MindTheGap [31] and Sealer [14] belong to the first category; all reads are indexed in a de Bruijn graph which is then traversed locally. On the opposite, GAPPadder [15], GapCloser [13] and GapFiller [32] recruit first a subset of reads based on mate anchoring of paired-end or mate-pair reads. GapCloser and GapFiller operate in an iterative manner, repeating the steps of recruitment and assembly until the gap is filled. The former tools using the whole read set have difficulty assembling repeat-rich sequences while the latter are limited in the gap size by the distance range between read mates. As a matter of fact, GAPPadder performs better with longer distance mate-pairs than with short-distance read pairs. Consequently, linked-read data that provide long-distance information, up to 30–70 Kb, are a promising source of data to improve local assembly results, as it can help identifying among the whole set of reads the ones that originate from the *locus* of interest.

Here, we present MTG-Link, a local assembly tool dedicated to linked-reads. The main feature of MTG-Link is that it takes advantage of the linked-read barcode information to get a subsample of reads of interest for the local assembly of each sequence. We demonstrate that linked-reads long-range information can substantially improve local assembly results on several linked-read datasets of large eukaryote genomes. We also show that it can be used for various local assembly use cases, such as gap-filling between scaffolds, characterization of clinically-relevant regions or reconstruction of Structural Variant alternative sequences.

## Implementation

MTG-Link performs local assemblies of specific *loci*, using linked-read data. For all use cases (specific *locus* assembly, intra-scaffold and inter-scaffold gap-fillings, as well as alternative allele reconstruction of large Structural Variants), the *locus* of interest is defined by two coordinates on a reference genome, indicating its left and right flanking sequences, and the sequence in-between to be assembled is referred to as the target sequence. Here, the reference genome corresponds to our genomic coordinate reference, regardless of its assembly quality or proximity to the re-sequenced individual.

The input of MTG-Link is a set of linked-reads, the target flanking sequences and coordinates in GFA format (genome graph format, with the flanking sequences identified as “segment” elements (S lines) and the targets identified as “gap” elements (G lines)) and an indexed BAM file obtained after mapping the linked-reads onto the reference genome. It outputs the set of assembled target sequences in Fasta format, as well as an assembly graph file in GFA format, complementing the input GFA file with the obtained assembled sequences.

**Fig. 1:**
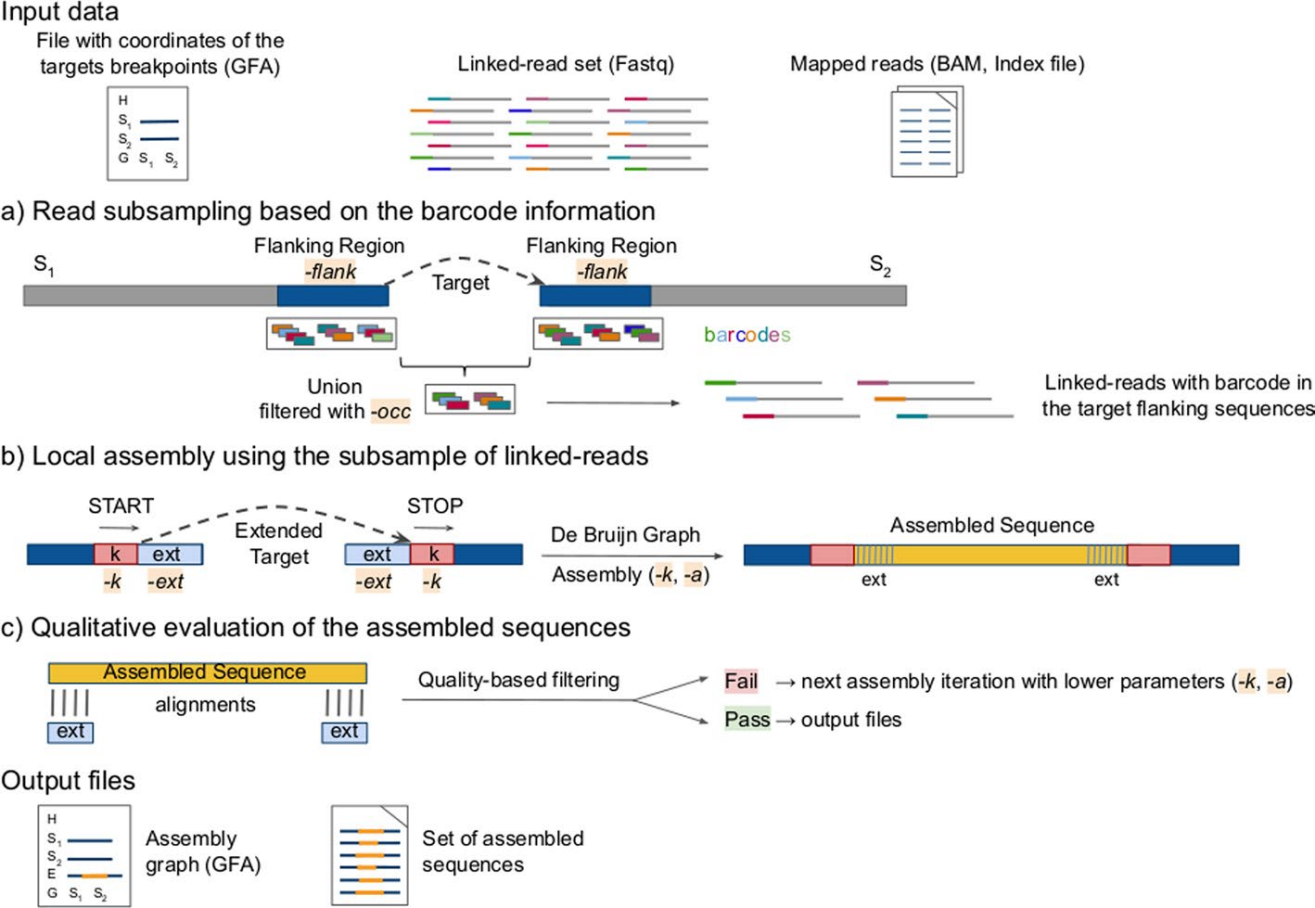
Overview of the MTG-Link pipeline. **a** Linked-reads whose barcode is observed in flanking regions surrounding the target sequence are extracted, and constitute the read subsample used in the local assembly step. **b** The local assembly is performed on an extended target, from the k-mer *START* (left) to the k-mer *STOP* (right), using the subsample of linked-reads obtained in **a**. **c** A quality score is assigned to the assembled sequence according to its alignment against the target flanking sequences. Only the assembled sequences with good quality scores are returned. Otherwise, a new assembly iteration is performed with lower de Bruijn graph parameters

In MTG-Link, each target sequence is processed independently in a three-steps process: (i) read subsampling using the barcode information of the linked-read dataset, (ii) local assembly by de Bruijn graph traversal and (iii) qualitative evaluation of the obtained assembled sequence. A schematic overview of the MTG-Link pipeline is shown in Fig. 1.

### Read subsampling

The purpose of the read subsampling step is to extract the linked-reads whose barcode is observed in flanking regions surrounding the target sequence (Fig. 1a).

First, a barcode list is computed from the reads mapped on both flanking sequences. This selection is performed based on two user-defined parameters, the flanking region size and the minimum number of occurrences in these regions for a barcode to be retained (-flank and -occ options). Then, the whole read file is searched for reads having the selected barcodes. In order to perform these two tasks efficiently given the large file sizes, we rely on the LRez toolkit [33] for barcode-based indexing and query of linked-read files.

### Local assembly

In a second step, the target is reconstructed with the set of selected reads, by searching one or more assembly paths between its left and right flanking sequences (Fig. 1b).

The local assembly is performed with the *fill* module of the software MindTheGap [31], which is based on a de Bruijn graph data structure to represent the set of input reads. Basically, starting from a k-mer *START*, it performs a breadth-first traversal of the de Bruijn graph, until the k-mer *STOP* is found or exploration limits are reached. It then enumerates all possible sequence paths between both k-mers. Notably, barcode information is not used in this step, and if several sequence paths exist in the graph, the algorithm does not seek to output two phased diploid sequences. In this case, all sequence paths are compared pairwisely and MindTheGap outputs a subset of representative sequences such that all sequences differ by more than 10% identity.

The choice of the input *START* and *STOP* k-mers is crucial for a successful traversal of the de Bruijn graph. Instead of extracting them from the reference genome sequence, which may diverge from the re-sequenced individual, the reads mapping at the corresponding coordinates are investigated to identify the most represented k-mers in the reads, ensuring these two kmers are actually existing nodes in the de Bruijn graph. In MindTheGap, as in any de Bruijn graph based assembler, two parameters have major impacts on the quality of the assembly: the k-mer size (-k) and the k-mer abundance threshold for including a k-mer in the graph (solid k-mer threshold -a). These parameters are usually set in accordance with the expected sequencing depth. In the case of MTG-link, the latter may vary depending on the efficiency of the barcode-based subsampling step. Hence for higher sensitivity, MTG-Link automatically tests different values for these two parameters until an assembled sequence is found.

### Qualitative evaluation and iterative assembly

For an evaluation purpose, the target sequence can be extended at both sides, such that the sequence to be assembled should overlap parts of the flanking sequences. The assembled sequences of these overlapping regions are useful to infer the quality of the whole assembled sequence, helping filtering out putative erroneous sequences. The evaluation is based on the alignment of these assembled sequences to their respective genome sequences, using Nucmer [34] (Fig. 1c). Only target’s assembled sequences with good extension alignments (more than 90% identity on more than 90% of the extension reference sequences) are returned. Otherwise, a new iteration of the local assembly step is performed with lower de Bruijn graph parameters.

### Implementation and availability

We provide an implementation of this method named MTG-Link, freely available at https://github.com/anne-gcd/MTG-Link under the GNU Affero GPL licence. MTG-Link is also available as a Bioconda package. MTG-Link is written in Python 3. The main steps are implemented in a modular way, allowing the user to start or re-run the program from previous intermediate results. As an example, the first step (read subsampling step) is not to be repeated if we want to perform the local assembly on the same input files but with different assembly parameters values. In order to speed up the process, the data handling and analysis are set up in a multi-threaded manner, thus allowing multiple target sequences to be processed simultaneously. Additional Python scripts for converting input and output files to the desirable formats are provided. Results shown here were obtained with release version v2.4.1. All experiments were performed on a cluster node equipped with 250 GB of RAM using 8 threads, on a 2.3 GHz CPU.

## Materials

### Linked-read datasets

#### stLFR *Homo sapiens* dataset

MTG-Link was applied on a human dataset obtained from the individual HG002 with the stLFR technology, from the Genome In A Bottle resources [20] (FTP links are given in Additional file 1: Section S1.1). After the removal of the PCR duplicates, this dataset is composed of approx. 1.5 billion 100 bp Illumina reads, with an effective read depth of 47X. We considered the assembly GRCh37 (hg19 version) as the human reference genome.

#### 10$\times$ Genomics *Heliconius numata* datasets

MTG-Link was also applied on linked-read datasets obtained from 12 individual genomes of the butterfly *Heliconius numata*, with the 10$\times$ Chromium Genomics technology (BioProject PRJNA676017) [5]. The number of reads in each dataset is approx. 110 million, with an effective coverage ranging from 20$\times$ to 47$\times$. For each individual, we used as reference genomes their draft genome assemblies obtained with the Supernova assembler [23] and available under the same BioProject ID. Experiments on randomly selected *loci* were performed on individual 37 (read depth of 40$\times$).

### Evaluation on randomly selected *loci*

To evaluate the accuracy of MTG-Link, we performed experiments where the target sequence to be assembled was a sequence already present in the reference genome at a *locus* randomly sampled. In such cases, the true sequence to be assembled is known but it is not used by the assemblers. It is only used to compute quality metrics by comparing it to the assembler’s output. Four target sizes were tested (1, 5, 10 and 20 Kb) on 63 and 57 randomly selected *loci* respectively for the human stLFR dataset and the butterfly 10$\times$ Genomics dataset (individual 37). MTG-Link was applied on all of these targets, testing different flanking region sizes (5, 10 and 15 Kb).

Assembled sequences were aligned to their reference sequence using Blastn [35]. The assembled sequences having more than 90% identity and coverage with the reference sequence were labelled as “successful”, otherwise they were considered as “erroneous”. The “no assembly” represented those for which no solution was returned. The success rate was then measured as the number of “successful” assemblies over the total number of target sequences that must be reconstructed. The assembly accuracy of the method was assessed as the number of “successful” assemblies over the number of assembled sequences (“successful” and “erroneous”).

We compared MTG-Link to several short-read local assemblers. MindTheGap [31], ABYSS-Sealer [14] and GAPPadder [15] were tested using the same evaluation protocol (command lines are given in Additional file 1: Section S1.2).

### Real use case: characterization of dark regions in clinically-relevant human genes

MTG-Link was applied on the reconstruction of dark regions in clinically-relevant human genes, using the stLFR *Homo sapiens* dataset. At this end, all dark regions listed by Ebbert et al. [36] and enclosed within the locations of 10 genes with known disease associations (ARX, NEB, TBX1, RPGR, HBA2, CR1, SMN1, SMN2, HSPA1A, HSPA1B), extracted from the GRCh37 annotation (hg19), were retrieved. This resulted in a set of 87 dark regions, of size between 21 bp and 8,043 bp, with a median and average size of 236 and 1,150 bp respectively. This set of dark regions was split into two types: 7 “dark by depth” regions and 80 “dark by mapping quality” regions. MTG-Link was applied on these two sets of regions with the HG002 stLFR sequencing dataset with default parameters. Results were evaluated with the same evaluation protocol as for randomly selected *loci*.

### Real use case: reconstruction of Structural Variant alternative sequences

MTG-Link was used to reconstruct the alternative sequences of large Structural Variants using the stLFR *Homo sapiens* dataset. From the gold standard SV callset of Genome In A Bottle obtained on the HG002 individual [37], we selected two sets of variants: one with deletions and one with insertions.

For the deletion set, we selected all deletion calls of size between 1 and 5 Kb, with a homozygous for the alternative allele (1/1) genotype and annotated as “SIMPLEDEL”. This resulted in a set of 121 deletion calls. For each deletion call, we attempted to reconstruct its breakpoint sequence by giving to MTG-Link as target locus coordinates the positions of the deleted segment on the reference genome expanded by 50 bp on each side. The expected size of the breakpoint sequence to be assembled was therefore 100 bp.

For the insertion set, we selected all insertion calls that were larger than 250 bp and annotated as “novel insertion” in the Delage et al. study [38]. This resulted in a set of 151 insertion calls, ranging in insertion size from 250 bp to 27,920 bp, and consisting of 104 insertions with a homozygous genotype and 47 insertions with a heterozygous genotype in HG002. The repeated nature of the context of the insertion site was retrieved from the Delage et al. study [38]. As the reported position of the insertion site may be imprecise, we attempted to reconstruct the insertion sequence by including 50 bp on either side of the insertion site. As the insertion sequences are known for this dataset [37], we were able to assess the accuracy of MTG-Link assembled sequences with the same evaluation protocol as for randomly selected *loci*.

### Real use case: inter-scaffold gap-filling of a *locus* of interest

We also applied MTG-Link to improve the contiguity of the Supergene P *locus* (1.3 Mbp) of the butterfly *H. numata* of 8 among 12 re-sequenced individuals [5]. Indeed, the Supergene P *locus* was previously reconstructed as a single scaffold for only four individual genomes, while the sequence of the *locus* was fragmented into several scaffolds (58 gaps in total) for the other 8 individuals. For these latter, we started by ordering the large scaffolds identified as belonging to the *locus* (BLAST comparisons with the related species *Heliconius melpomene*, personal communication from Paul Jay [5]) using the number of their common barcodes calculated by LRez [33]. In a second step, we performed gap-filling on the 58 candidate pairs with MTG-Link.

## Results

### Assessing MTG-Link results on evaluation datasets

#### Assembly of randomly selected *loci*

**Fig. 2:**
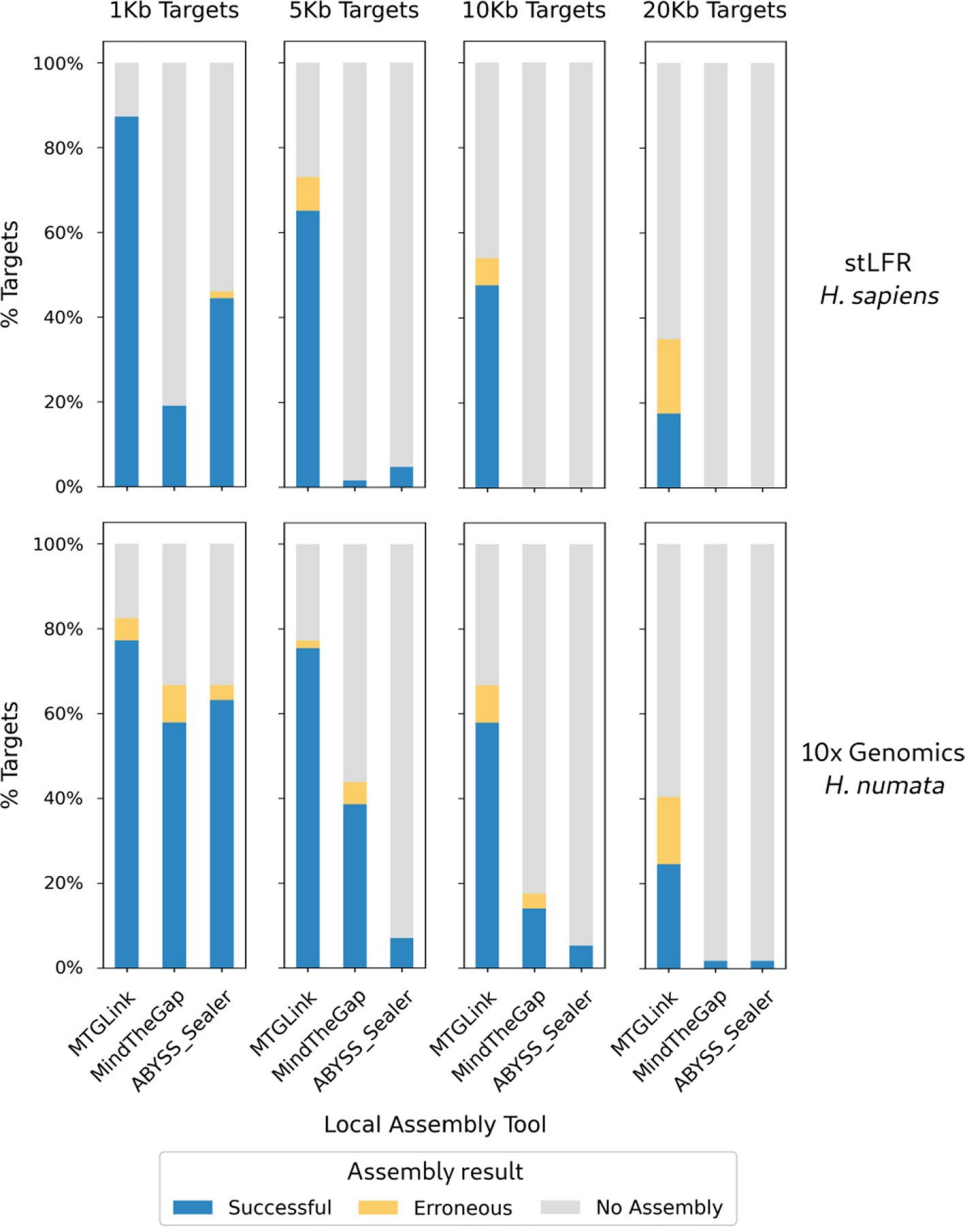
Comparison of assembly results for several local assembly tools and several target sizes. Results are shown for the stLFR *H. sapiens* dataset (top) and for the 10$\times$ Genomics *H. numata* dataset (bottom). MTG-Link, MindTheGap and ABYSS-Sealer were applied on four sets with different target sizes (1, 5, 10 and 20 Kb), each composed of 63 and 57 randomly selected *loci* resp. for the *H. sapiens* and the *H. numata* datasets

MTG-Link was first applied on several evaluation datasets, composed of real linked-read data (an stLFR *H. sapiens* dataset and a 10$\times$ Genomics *H. numata* dataset) and randomly selected *loci* for which the sequence to assemble is known, to precisely estimate its success rate and assembly accuracy. The results obtained with MTG-Link for several target size (1, 5, 10 and 20 Kb) are presented in Fig. 2.

For small targets (1 Kb), MTG-Link returns an assembly for most targets with a good accuracy. Notably, for the human dataset, 87.3% of the 1 Kb targets are successfully assembled (success rate) and MTG-Link did not return any incorrect assemblies (e.g. with more than 10% of sequence or size divergence with the reference sequence), giving an assembly accuracy of 100%. Assembled sequences are highly similar to their target sequence in the reference genome with an average of 99.8% identity. However, success rate and assembly accuracy metrics decrease as the target size increases. The success rate falls below 50% for 10 Kb and 20 Kb targets. The assembly accuracy remains high for 5 Kb and 10 Kb targets (89.1% and 88.2% resp.), but drops to 50% for the largest target size (20 Kb) (top part of Fig. 2). When using a more (95%) or less (85%) stringent percentage identity threshold to consider an assembled sequence as accurate, assembly metrics remain almost unchanged (Additional file 1: Table S1).

A similar trend is observed for the 10$\times$ Genomics *H. numata* dataset, but with a smaller decrease in performance with the target size, as the success rate remains high for 1 to 10 Kb targets (70.2% for all three sizes combined, bottom part of Fig. 2).

**Fig. 3:**
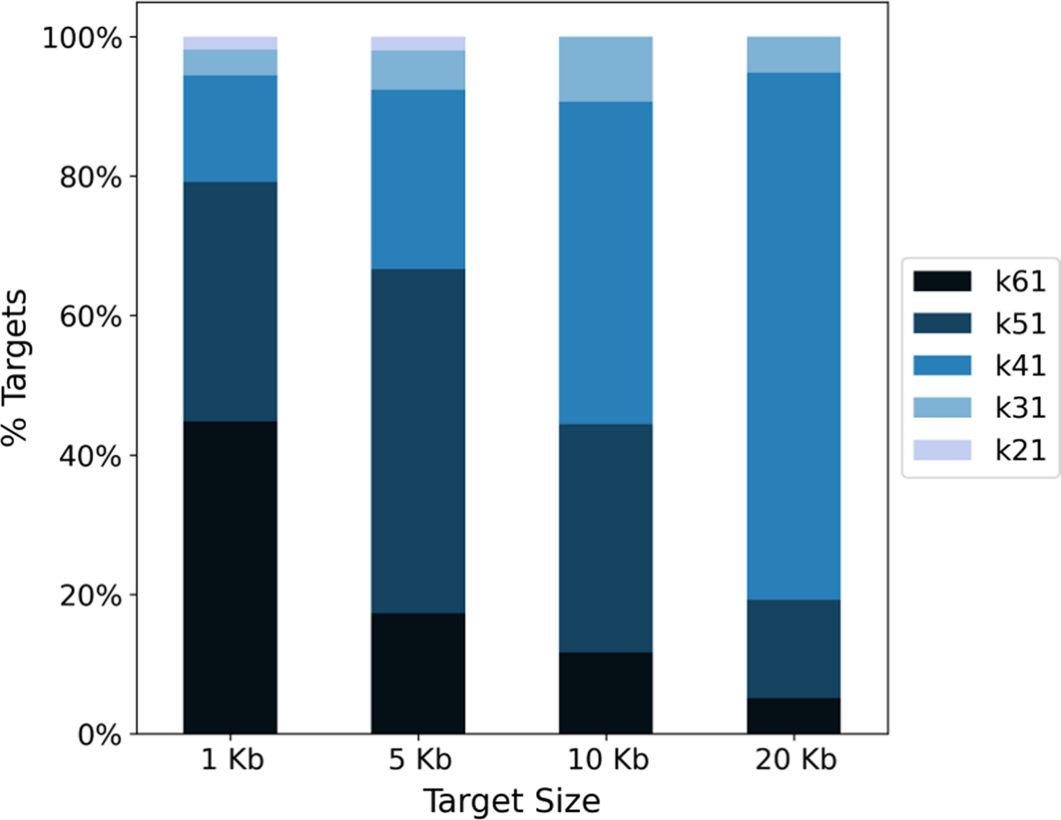
Proportion of k-mer sizes used by MTG-Link to assemble the successful assemblies depending on the target size. MTG-Link was run on the stLFR *H. sapiens* dataset with the parameter -k ranging from 61 to 21 with intervals of 10

Analyzing the successful assemblies, we can observe that they were obtained with different k-mer sizes (-k) depending on the target size (Fig. 3). We recall that MTG-Link automatically tests several k-mer sizes in decreasing order, and stops when a good quality assembly is obtained. We can see in Fig. 3 that most 1 to 5 Kb targets are successfully assembled at the first assembly attempts, e.g. for -k equals 61 or 51. As the target size increases, the assemblies are obtained with smaller -k values, meaning that assembly with larger -k values failed.

#### Barcode-based subsampling analysis

**Fig. 4:**
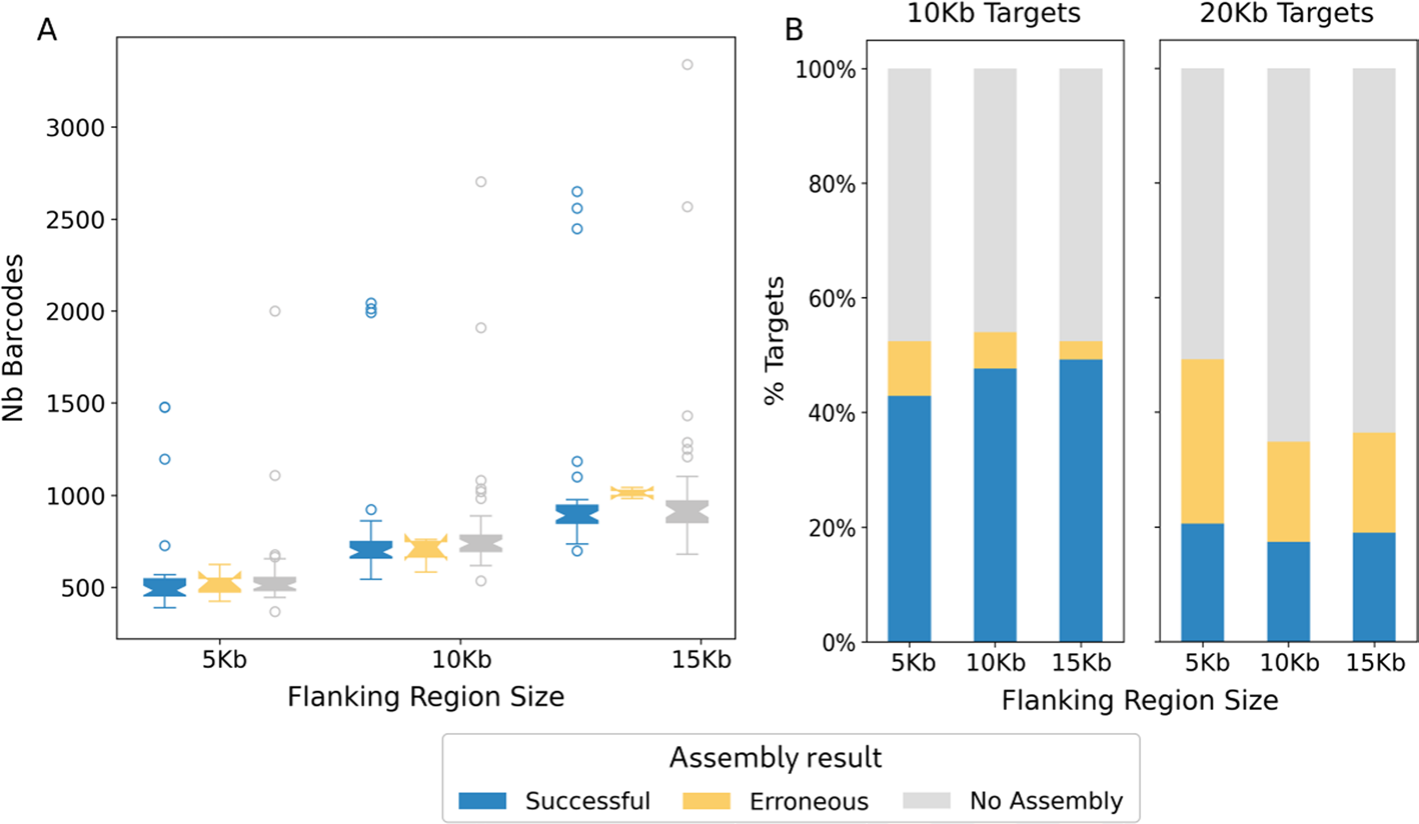
Influence of the flanking region size on the assembly quality. MTG-Link was run on the stLFR *H. sapiens* dataset with varying flanking region size (-flank parameter): 5 Kb, 10 Kb, 15 Kb. **A** Correlation between the flanking region size and the number of barcodes selected, and influence of these two parameters on the assembly quality for targets of 10 Kb. **B** Influence of the flanking region size on the assembly quality for targets of 10 Kb and 20 Kb

The main feature of MTG-Link is its read subsampling step based on the barcode information of the linked-reads. For all the combined target sizes of the stLFR *H. sapiens* dataset, 855 barcodes were selected on average from the target flanking regions, resulting on average to 111,905 reads given as input for the *de novo* assembly. The number of selected barcodes is quite variable between the targets, with for instance a standard deviation of 407 barcodes for the 63 10Kb targets (Fig. 4A). However, the number of selected barcodes seems not to be directly linked to the assembly success as the missing and erroneous assemblies do not show either much less or much more selected barcodes (Fig. 4A). Similarly, the distributions of number of selected reads are similar between the three assembly result status, as also shown by the high correlation between the number of selected barcodes and the number of reads extracted from this selection (Additional file 1: Fig. S1).

In MTG-Link, the main parameter governing the barcode selection is the size of considered flanking regions (parameter -flank). Indeed, when increasing this parameter, the number of selected barcodes increases but it does not impact substantially the assembly performances (Fig. 4A, B; see also Additional file 1: Fig. S2). In other words, even if we select more barcodes to increase the size of the subsampled read sets, the success rate and the assembly accuracy do not increase.

### Comparison with other approaches

To our knowledge, no other reference-free local assembly tool using linked-read data has been developed. Therefore, MTG-Link was compared to short-read local assemblers that can not take advantage of the barcode information of the linked-reads. This comparison is therefore meant to highlight the added value of this type of data. MTG-Link was first compared to MindTheGap [31], as this is the tool used in the local assembly step of our method, thus enabling to evaluate the benefit of the read subsampling step. MTG-Link was also compared to the most recent short-read local assemblers: ABYSS-Sealer [14] and GAPPadder [15]. However, we did not manage to make GAPPadder work on our datasets as it is no longer maintained, so we will present only the results obtained with ABYSS-Sealer.

For all target sizes, MTG-Link outputs more successful assemblies with a better assembly accuracy than the two short-read local assemblers MindTheGap and ABYSS-Sealer (Fig. 2). On the stLFR *H. sapiens* dataset, for all target sizes combined, MTG-Link shows a success rate of 54.4%, much better than the success rates of 5.2% and 12.3% for MindTheGap and ABYSS-Sealer respectively. Besides, the differences tend to increase with the target size. In particular, no target over 10 Kb could be assembled by any of the two short-read local assemblers MindTheGap and ABYSS-Sealer. Therefore, MTG-Link outperforms these two tools. Similar results were obtained for the 10$\times$ Genomics *H. numata* dataset (see also Additional file 1: Table S1 for results with varying sequence similarity thresholds).

The computational performances (speed and memory usage) of MTG-Link are of the same order of magnitude of those of the short-read local assemblers MindTheGap and ABYSS-Sealer (Additional file 1: Tables S2 and S3).

### Application of MTG-Link to the characterization of dark regions in clinically-relevant human genes

Some regions in the human genome, referred to as *dark regions*, remain inaccessible to standard short-read sequencing technologies, either due to insufficient sequencing depth (“dark by depth” regions with $\le$5 aligned reads) or to low mapping quality (“dark by mapping quality” regions with $\ge$90% of aligned reads having a mapping quality (MAPQ) <10) [36]. In the Ebbert et al. study [36], they highlighted ten human genes that may be relevant to human health and disease and that contain such dark regions (7 “dark by depth” and 80 “dark by mapping quality”). We tested here if MTG-Link could help in characterizing these sequences that are inaccessible by standard short-read sequencing.

MTG-Link returns an assembled sequence for 4 regions out of the 7 “dark by depth” regions (57.1%), with an assembly accuracy of 100%. All assembled regions have a size of less than 100bp. This shows that at least some of these regions are sufficiently sequenced with the stLFR technology and can be recovered through local assembly.

On the “dark by mapping quality” region set, MTG-Link has a success rate of 72.5% and an assembly accuracy of 100%. Considering only the regions smaller than 5 Kb, we obtain very good results with a success rate of 79.2%. These “dark by mapping quality” regions arise principally from duplicated genomic regions. These results show that by exploiting their less repeated flanking regions and the long-range information in linked-reads, MTG-Link is able to characterize these sequences, contrary to standard short-read mapping approaches.

### Application of MTG-Link to the reconstruction of Structural Variant alternative sequences

We applied MTG-Link to reconstruct the alternative sequences of known large Structural Variants, namely insertions and deletions, in human HG002. From the gold standard SV callset provided by the Genome In A Bottle consortium on the HG002 individual [37], we selected a subset of 121 deletion calls of size between 1 and 5 Kb and all novel insertion calls that were larger than 250 bp (N=151).

For deletions, the sequence of the alternative allele is simply the adjacency of both sequences at each side of the deleted sequence in the reference genome. We tried to reconstruct these breakpoint sequences by defining the target *loci* as the deletion segments extended by 50 bp on either side, resulting in 100 bp breakpoint sequences to be assembled. Among the 121 deletions, MTG-Link reports an assembled sequence for 109 (90%), all of which of size much smaller than the deletion size and close to the expected breakpoint size of 100 pb (median size of 100 bp and maximal size of 116 bp). These assemblies represent a validation of these deletion calls by confirming the absence of the deleted sequence at these *loci* in the HG002 individual.

**Fig. 5:**
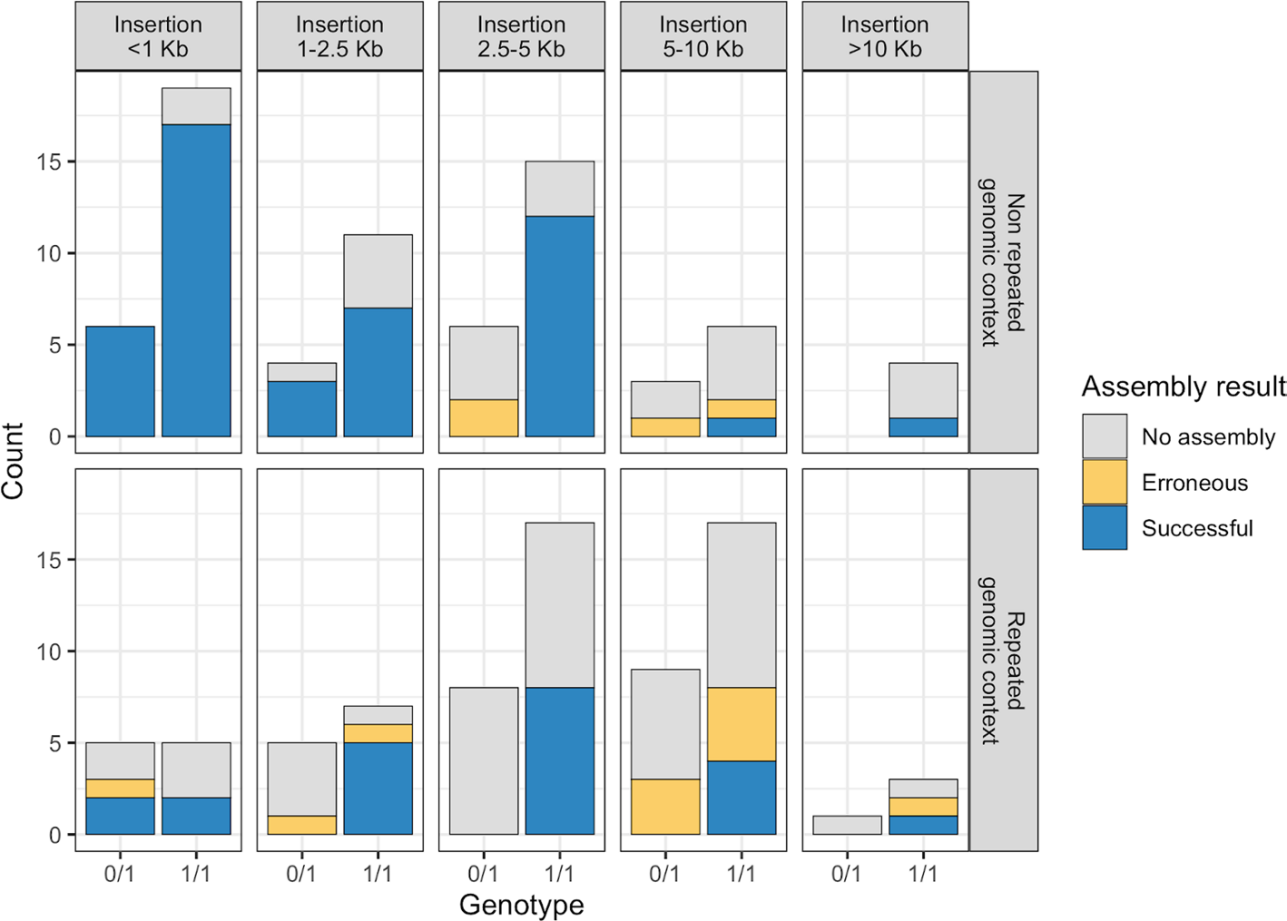
Results of MTG-Link on the reconstruction of human insertion variants. MTG-Link was run on 151 insertion calls with the stLFR *H. sapiens* dataset. The results are categorized by the insertion size, the variant genotype in individual HG002 (0/1 and 1/1 for heterozygous and homozygous insertions resp.) and the repeated nature of the genomic context of the insertion site

On the contrary, for insertions, the characterization of the alternative alleles is a more difficult task. They are much larger sequences to assemble as they are the whole inserted sequences and, in the case here of novel insertions, they are sequences absent from the reference genome. All insertion sequences are resolved in this dataset, as they were called mainly with long-read sequencing data. Therefore, we were able to evaluate the assembled sequences obtained by MTG-Link and assess the sensitivity and accuracy of the method with respect to various insertion features (size, genotype, repeat context of insertion site). The results for the 151 insertions are presented in Fig. 5. MTG-Link has a success rate of 45.7% and an assembly accuracy of 82.1%. We observe that, in addition to the size of the sequence (> 5 Kb), the assembly performance of MTG-Link is directly related to the presence of repeated elements around the insertion site as well as to the insertion genotype. Indeed, the expected read depth of the inserted sequence for the heterozygous variants is half the genome-wide read depth and makes the *de novo* assembly more difficult. At least one of these three factors is reported in 86.6% of the non-assembled insertions and 100% of the erroneously assembled insertions. Considering only the homozygous insertions smaller than 5 Kb (N= 74 insertions), MTG-Link obtains very good results with a success rate of 68.9% and an accuracy of 98.1%.

### Application of MTG-Link to improve the contiguity of the Supergene P *locus* in *H. numata* individuals

We applied MTG-Link on inter-scaffold gaps to improve the contiguity of the Supergene P *locus* of the butterfly *H. numata* in eight individuals. For each of these eight individuals, we attempted to fill the gaps between the scaffolds using MTG-Link. We succeeded in reducing the number of scaffolds in the Supergene P *locus* for all *H. numata* individuals. For two of them (individuals 28 and 30), the Supergene P *locus* was reconstructed as a single scaffold in one step of gap-filling. For the others, the assembly was still fragmented and we performed additional steps of extra contigs recruitment. We retrieved additional small scaffolds that shared at least 50 barcodes with the initial set of scaffolds. Finally, we succeeded in filling 45 out of the 58 initial gaps with MTG-Link (Additional file 1: Table S4).

## Discussion and conclusion

We provide a novel tool for local assembly that is dedicated to linked-read sequencing data. We validated this tool on several datasets, composed of real linked-read data and randomly selected *loci* for which the sequence to assemble is known, to precisely estimate its success rate and assembly accuracy. The results described above show that our method can be applied on different organisms, as well as on different linked-read sequencing technologies, to obtain better assemblies than with other existing local assemblers. The results presented here were obtained with the two linked-read technologies that are the most represented in terms of datasets in public repositories, namely 10$\times$ Chromium Genomics and stLFR technologies. But, our method is also compatible with Tell-Seq and Haplotagging technologies. Several parameters in MTG-Link, such as the flanking region size or the minimal number of occurrences of a barcode in flanking regions for a barcode to be selected, allow our tool to adapt to technology specificities such as the density of barcodes or the size of the long molecules. Another feature that may differ between these technologies and may impact the assembly success is the sequencing biases with respect to GC content, as it has already been observed that some high GC content regions have poor or no coverage in linked-read data [21, 39]. Finally, with Haplotagging datasets, the assembly success will likely depend on the level of sequence diversity in the dataset, since this technology allows the simultaneous sequencing of many individuals but each at very low coverage.

To our knowledge, this is the first local assembly tool dedicated to linked-read data that does not need a reference sequence to guide the assembly. We have therefore compared our tool MTG-Link to generic short-read local assembly tools that could not use the long-range information given by each read barcode. In particular, we compared MTG-Link to MindTheGap, which is one of the component of the MTG-Link pipeline. As both rely on the same assembly algorithm, we could assess the benefit of the main feature of MTG-Link: its barcode-based read subsampling step prior to local assembly. The much better results obtained by MTG-Link compared to using solely MindTheGap demonstrate that the long-range information contained in linked-reads allows improving substantially local assembly qualities. Contrary to MindTheGap which builds its de Bruin graph with the whole read set, the read subsampling step of MTG-Link allows the enrichment of reads originating from the target *locus* in the read set used for the assembly. By discarding a large fraction of reads originating from other regions of the genome, we reduce the noise and complexity in the assembly graph, thus making the search for the target sequence path easier and achievable in a reasonable time.

Our results show that MTG-Link is able to assemble successfully large sequences, up to dozens of Kb. As expected, we observed that the smaller the target sequence the better the results. Actually, the risk of the read subsampling approach is to decrease the read depth on some parts of the target sequence and to disconnect the assembly graph, and this risk increases with the size of the target. This is illustrated by the observed relationship between the successful k-mer value used for the assembly and the target size (Fig. 3). The larger the target size, the more the assembly fails with large k-mer values. De Bruijn graph traversal is known to be easier and to give more accurate paths with large k-mer values, as long as the read depth is sufficient for the graph to be connected. Hence, these results show that the read depth may be insufficient on some parts of the longer targets, probably in the middle where the distance to the flanking sequences is the greatest. Indeed, barcode-based read recruitment is limited by the size of the long DNA molecules obtained during the linked-read library preparation. As a matter of fact, increasing the flanking region size did not improve the results, since barcodes of interest are more likely to be found near the target sequence than far away. The target size limit is thus likely to depend on the size distribution of the long DNA molecules. Here, for molecules of average size around 55 Kb (around 70 Kb and 40 Kb for the stLFR *H. sapiens* and the 10$\times$ Genomics *H. numata* datasets respectively), MTG-link was still able to successfully assemble around 20% of the 20 Kb target sequences. Decreasing the k-mer value still allows the assembly of some of the larger targets and Fig. 3 shows that there is not a single k-mer size that outperforms the others for any target size; hence the importance of testing several k-mer sizes to optimize assembly results. This is performed automatically and efficiently in MTG-Link thanks to the last step of the pipeline, the qualitative evaluation of each obtained assembled sequence.

The second feature impacting the performances of MTG-Link is typical to any *de novo* assembly tool: it is the presence of repeats in or around the target sequence. This is particularly illustrated when comparing the results obtained for the assembly of insertion variants in the human HG002 individual, whether the insertion site is located in a repeated region or not (Fig. 5). Notably, this repeated feature impacts both the success rate and the accuracy of the method. As a matter of fact, among the non-assembled 10 Kb targets of the randomly selected *loci* of the human dataset, 30% were actually assembled successfully by MTG-Link but returned among other possible solutions. These multiple solutions have at least 10% divergence between them, and the multiple nature of the solutions reflects the repetitive nature of the region to be assembled. As it is not possible to select the correct assembly among all the multiple solutions, MTG-Link does not return any of them by default. Concerning the assembly accuracy, we observed that many erroneous assemblies showed high sequence similarities with the reference sequence, but were incomplete. In several cases, we observed the presence of direct repeats in the reference sequence, generating a cycle in the de Bruijn graph whose sequence (between repeat copies) is lost in the assembly. This may explain why some targets are erroneously assembled with MTG-Link and why the accuracy decreases with the target size since the likelihood of harbouring at least two repeat copies increases with the size of the sequence.

Even if some targets are particularly difficult to assemble, we proved the usefulness of MTG-Link in three biological applications, namely to validate and sequence-resolve Structural Variant calls, to reconstruct some repetitive regions in clinically-relevant genes and to improve the contiguity of a *locus* split into several scaffolds in an initial assembly. Large insertion variants are one of the most difficult structural variant types to discover and fully characterize in re-sequencing datasets [40], because the alternative allele, the inserted sequence, is most often not contained in read mapping results. In particular, many short-read SV callers can predict insertion site locations on the reference genome but are not able to resolve the whole inserted sequence [38]. Our results show that MTG-Link could be combined to short-read SV callers to improve their characterization of insertion variant calls. Similarly, for other SV types, we have shown that MTG-Link can be used for the local assembly of deletion breakpoints. This experiment on deletions is a proof of concept also for other SV types, such as inversions and translocations, since their breakpoint sequences are defined similarly to deletion breakpoints, e.g. by a sequence adjacency between two sequences that are already present in the reference genome. MTG-Link could therefore be used as a post-processing tool after SV calling, helping to validate SV calls or to filter out false positive calls. The third presented application can be referred to as gap-filling in a draft assembly composed of several contigs or scaffolds. Linked-read data have been massively produced to generate draft genome or haplotype assemblies. As for the Supergene P *locus* in *H. numata*, this is quite frequent that some *loci* of interest have already been identified in the studied organism and we showed that MTG-Link is a useful tool to help analyze and characterize these *loci* in several individuals.

## Supplementary Information

**Additional file 1.** Additional information, figures and tables.

## Data Availability

The source code of MTG-Link is available at https://github.com/anne-gcd/MTG-Link under the GNU Affero GPL licence. MTG-Link is also available as a Bioconda package. Project name: MTG-Link. Project home page: https://github.com/anne-gcd/MTG-Link. Operating system(s): Platform independent. Programming language: Python. Other requirements: Biopython 1.79, Gfapy 1.2.0, Mummer 3.23, Pathos 0.2.8, Pysam 0.19.1, Regex, LRez 2.2.3 or higher, MindTheGap 2.2.3 or higher. License: GNU Affero GPL licence. Any restrictions to use by non-academics: licence needed

## Abbreviations

GFA: Graphical Fragment Assembly
BAM: Binary Alignment Map
PCR: Polymerase chain reaction
SV: Structural Variant
BLAST: Basic Local Alignment Search Tool
